# The effect of financial inclusion on open defecation and sharing of toilet facilities among households in Ghana

**DOI:** 10.1371/journal.pone.0264187

**Published:** 2022-03-04

**Authors:** Mustapha Immurana, Kwame Godsway Kisseih, Hadrat Mohammed Yusif, Ziblilla Mbanba Yakubu

**Affiliations:** 1 Institute of Health Research, University of Health and Allied Sciences, Ho, Ghana; 2 Christian Health Association of Ghana Secretariat, Accra, Ghana; 3 Department of Economics, Kwame Nkrumah University of Science and Technology, PMB, Kumasi, Ghana; 4 Department of Applied Economics, University for Development Studies, Tamale, Ghana; Cranfield University, UNITED KINGDOM

## Abstract

Globally, and in Ghana, a lot of people do practice open defecation as well as share toilet facilities with other households. Meanwhile, open defecation in particular, is associated with numerous negative health and economic effects. To this end, a number of empirical studies have been conducted on the determinants of access to sanitation facilities among households in Ghana. Nonetheless, while financial inclusion (sustainable ways of ensuring easier accessibility to cheap and useful financial products and services among individuals/firms) can enhance the ability of households or individuals to afford toilet facilities, hence, could help in curbing open defecation and sharing of toilet facilities among households, the previous studies on Ghana did not pay attention to it. This study therefore uses data from the 7^th^ round of the Ghana Living Standards Survey (GLSS7) to examine the association of financial inclusion with open defecation and sharing of toilet facilities among households in Ghana. The binary logit regression is used as the empirical estimation technique. The results show that, financial inclusion in general is associated with lesser likelihood of open defecation and sharing of toilet facilities among households in Ghana after controlling for welfare quintile, urban or rural residence and other covariates. Moreover, while informal financial inclusion is statistically insignificant, formal financial inclusion is found to be associated with reduced open defecation and sharing of toilet facilities among households. Thus, in the attempt to eliminate open defecation as well as reduce the sharing of toilet facilities among households in Ghana, conscious efforts should be devoted towards enhancing formal financial inclusion.

## Introduction

Poor or inadequate sanitation is a challenge confronting the world. This is because, globally, inadequate sanitation is responsible for 432 000 diarrhoeal deaths on a yearly basis. Typhoid, hepatitis A, dysentery and some neglected tropical diseases including schistosomiasis are linked to poor sanitation. Thus, given that poor sanitation practices such as open defecation are widespread in countries with the highest levels of poverty, wealth disparities and malnutrition, they perpetuate a vicious cycle of poverty and disease, hence reducing human wellbeing [1].

In spite of the negative health effects of poor sanitation, 494 million people in the world practice open defecation [2]. Even among households who use toilet facilities, although sharing a toilet facility with another household is considered less hygienic [3], a number of them share these facilities.

In Ghana, the story is not different. For instance, as of 2020, 18% and 32% of the total population and rural dwellers, respectively, practiced open defecation [2]. It is therefore not surprising that no district in Ghana has achieved an open defecation free status [4]. Also, each year, open defecation costs Ghana $79 million, while roughly, 19000 Ghanaians die from diarrhoea mainly because of poor water, sanitation and hygiene [5].

A major factor that can influence access to sanitation facilities is financial inclusion (sustainable ways of ensuring easier accessibility to cheap and useful financial products and services among individuals/firms [6]). It is therefore not farfetched that, financial inclusion is regarded as a vehicle towards attaining a number of the Sustainable Development Goals (SDGs) given its role in making financial resources easily available to households/individuals as well as firms, hence positioning them better to enjoy livelihoods that are sustainable [6,7]. Thus, financial inclusion can help households to acquire toilet facilities by providing easy access to financial resources. This can therefore aid in reducing open defecation and sharing of toilet facilities among households.

Nonetheless, to the best of our knowledge, majority of the studies that have examined the determinants of open defecation or access to sanitation facilities did not pay attention to the role of financial inclusion [8–20]. Only a few studies have reported the role of financial inclusion in enhancing access to sanitation facilities [21–25]. For instance, Pories [21] found in India that, financial inclusion that enhanced access to toilet facilities at home, enabled at least a household member to devote the time previously wasted on defecation towards an economic activity. Bhandarkar [22] reported that the provision of financial inclusion (small loans) has ensured access to toilet facilities at home for millions of women and girls in India. Similarly, informal financial inclusion made available by a women association in Nigeria has been found to enhance access to sanitation facilities among households [23]. In addition, the provision of sanitation loans (among other factors) has been found to decrease open defecation as well as enhance access to improved sanitation facilities in Kenya [24].

While none of the above studies was conducted on Ghana, they also did not examine the association between an incremental level of financial inclusion (switching from no financial inclusion to informal financial inclusion to formal financial inclusion) and access to sanitation facilities. Nonetheless, doing so is very relevant for policy purposes as it will reveal which form of financial inclusion (formal and informal) should be given the needed attention in the attempts to oust open defecation and reduce sharing of toilet facilities among households. To this end, this study investigates the relationships between financial inclusion, and open defecation as well as sharing of toilet facilities among households in Ghana. In doing so, the study examines how an incremental level of financial inclusion is associated with open defecation and sharing of toilet facilities among households in Ghana, making it the first of its kind, to the best of our knowledge. The findings of this study, therefore help in highlighting as to which form of financial inclusion (formal or informal) should be used as a strategy towards enhancing access to sanitation services in Ghana, hence, aid in attaining the SDGs that are directly or indirectly linked to an improvement in access to sanitation services.

## Materials and methods

### Data

This study uses data from the 7^th^ round of the Ghana Living Standards Survey (GLSS7). The GLSS7 was a nationwide cross-sectional data collected from October, 2016 to October, 2017 using a two-stage stratified sampling technique. The primary sampling units were made up of 1000 enumeration areas. Using probability proportional to size, the primary sampling units were assigned to the 10 regions in Ghana (at the time of the data collection, the regions in Ghana had not been increased to 16). The enumeration areas were further divided into rural and urban areas. The secondary sampling units were arrived at through a complete listing of households in the selected primary sampling units. A sample size of 15000 households was arrived at by systematically selecting 15 households from each primary sampling unit. In the end, out of the 15000 households selected, 14009 were successfully interviewed [26]. It must be stressed that the analyses done in the present study are not restricted to only household heads but all individuals in the household with complete information for all included variables. Thus, apart from sub-sample analyses, all full sample analyses exceed the sample size of 14009. Specifically, we arrive at a full sample of 36433 and 16562 individuals for the open defecation and sharing of toilet facilities models respectively.

### Variables and estimation technique

In this study the dependent variables are open defecation and sharing a toilet facility with other households. With regard to open defecation, the survey asked a question about the type of toilet facility used. So, for the purposes of this study, if a household does not use any toilet facility (field, bush, beach etc.) it is coded as one (1) and if otherwise (uses a toilet facility (pit latrine, water closet, public toilet etc.)) it is coded as zero (0). Concerning the second dependent variable, if a household shares a toilet facility with other household(s), it is coded as one (1) and if otherwise (does not share), it is coded as zero (0).

The main independent variable is financial inclusion. We measure financial inclusion in three ways: i) financial inclusion (combined financial inclusion (both informal and formal financial inclusion)): thus, whether an individual has a bank account or contributes to a formal or informal savings and loans scheme (yes or no), ii) informal financial inclusion: whether a person has a traditional savings and loans account called susu (yes or no) and iii) formal financial inclusion: whether a person owns at least one of the following accounts; commercial bank, mortgage, rural bank, formal savings and loans scheme and credit union (yes or no). We do not add mobile money account to our formal financial inclusion measure (hence the combined financial inclusion measure) because, while it involves very less stringent requirements to acquire, to the best of our knowledge, mobile money in Ghana does not provide financial credit. Nonetheless, all our formal and informal financial inclusion measures provide financial credit. Also, we drop the variable capturing ‘other forms’ of financial institutions because it is made up of both formal and informal financial institutions that cannot be separated. Moreover, most of the formal and informal financial institutions stated in that variable (‘other forms’ of financial institutions) have already been captured by our financial inclusion indicators.

Therefore, we run four models, i) with the combined financial inclusion measure relative to those without financial inclusion (Panel A), ii) those with informal financial inclusion relative to those without financial inclusion (Panel B), iii) individuals with formal financial inclusion relative to those without financial inclusion (Panel C) and iv) persons with formal financial inclusion compared with those with informal financial inclusion (Panel D). Doing so helps in examining how incremental levels of financial inclusion are associated with open defecation and sharing of toilet facilities.

The other independent variables include sex, educational qualification, region, religion, residence (urban/rural), rent agreement (holding agreement of dwelling), national welfare quintile, total expenditure per day per adult and household size. These variables have been used by past studies [see 9,13,14,18–20]. Theoretically, the choice of welfare quintile, total expenditure per day per adult and education is justified based on the human capital model of demand for health by Grossman [27] which posits that, income (expenditure) and education are major determinants of demand for health inputs (sanitation in the case of this study). Hence, the existence of income/welfare inequalities between urban and rural settings as well as regions, justify the use of residence and region as covariates. Also, rent agreement is used because individuals who own their dwellings are more capable of having the financial resource to own toilet facilities relative to those who don’t own their dwellings. Moreover, among those who don’t own their dwellings, even if they are capable of acquiring toilet facilities, may need the permission of their landlords/landladies to do so.

Also, it has been found that, for religious reasons, some people may find it inappropriate to acquire toilets in their houses because they will be offending their gods, while females may opt for open defecation relative to the use of public toilets for privacy [10]. Also, in order to avoid inconvenience, rising size of households may push them to acquire their own toilet facilities instead of sharing with other households.

Notwithstanding the above, we are unable to include specific cultural practices as variables in our model because they are not found in the dataset. However, religion, residence type and region, have links with certain cultural practices, hence they serve as good proxies.

It should be noted that education, religion and rent agreement are recoded from their original nature for the purposes of this study. All the independent variables are categorical (hence treated as dummy variables) except total expenditure per day per adult and household size that are continuous.

Given the dichotomous nature of the dependent variables, this study employs the binary logit regression as the estimation technique [28]. All the binary logit regression results are reported using Odds Ratios (ORs) in order to find out the quantum of the associations between the dependent variables and the independent variables [29]. For robustness purposes, we use the binary probit regression as an additional estimation technique (see Appendix). Moreover, as recommended by the Ghana Statistical Service and ICF [30], we apply weights to all our variables (using the iweight, svy and svyset routines in STATA taking into account of clusters, regions and rural and urban settings) in order to make them regionally and nationally representative, hence, deal with any biasedness associated with the complex sampling frame used in collecting the data.

## Results

In this section, we present the descriptive statistics of the socio-demographic background of respondents as well as regression results of the associations between financial inclusion, and open defecation as well as sharing of toilet facilities among households in Ghana.

### Descriptive statistics

From the results (Table 1), we find that majority of the respondents are females (51.8%). Also, only 39% of the respondents have educational qualifications whiles most of them are Christians (74.2%) and rural dwellers (50.3%).

**Table 1:** Socio-demographic background of respondents.

Variable	Percent	
**FI1 (formal or informal financial inclusion)**		
Yes	20.6	
**FI2 (informal financial inclusion)**		
Yes	3.2	
**FI3 (formal financial inclusion)**		
Yes	18.7	
**Sex**		
Male	48.2	
Female	51.8	
**Educational qualification**		
Yes	39.0	
**Religion**		
No religion	3.5	
Christian	74.2	
Traditionalist/other	3.2	
Islam	19.1	
**Region**		
Western	10.7	
Central	7.8	
Greater Accra	16.0	
Volta	8.4	
Eastern	10.7	
Ashanti	18.8	
Brong-Ahafo	9.8	
Northern	10.7	
Upper East	4.1	
Upper West	3.0	
**Residence**		
Urban	49.7	
Rural	50.3	
**Open defecation**		
Yes	20.8	
**Share toilet facility with other households?**		
Yes	58.6	
**Rent agreement**		
Owning	54.2	
Renting	21.7	
Rent free	23.6	
Perching/squatting	0.4	
**National welfare quintile**		
First	21.0	
Second	20.5	
Third	19.9	
Fourth	19.6	
Fifth	19.0	
		**Mean**
Total expenditure per day per adult^a^		1.8
Household size		6.2

FI1: Combined financial inclusion (respondents with formal or informal financial inclusion); FI2: Respondents who have informal financial inclusion; FI3: Respondents who have formal financial inclusion;

a: Log of variable is used.

Source: Authors’ computation from GLSS7.

Also, the results show that only 20.6% of the respondents are financially included. Further, 20.8% and 58.6% of the respondents practice open defecation and share toilet facilities with other households respectively. The statistics of all other socio-demographic variables can be seen in Table 1.

### Regression results

In this sub-section, regression results of the associations between financial inclusion (combined, formal and informal), and open defecation (Table 2) as well as sharing of toilet facilities (Table 3) among households are presented. It must be stressed that in interpreting and discussing the results as well as making conclusions, we focus on only the adjusted estimates since they control for other covariates.

**Table 2:** Logistic regressions: Effect of financial inclusion on open defecation.

	Unadjusted ORs	(A) Adjusted ORs	Unadjusted ORs	(B)Adjusted ORs	Unadjusted ORs	(C) Adjusted ORs	Unadjusted ORs	(D) Adjusted ORs
**FI1 (Ref: No financial inclusion)**								
Yes	0.294***	0.747***						
	(0.0232)	(0.0686)						
**Sex (Ref: Female)**								
Male	1.000	1.018	1.077***	1.010	1.007	1.022	0.813**	1.257*
	(0.0264)	(0.0386)	(0.0307)	(0.0413)	(0.0278)	(0.0389)	(0.0688)	(0.168)
**Educational qualification (Ref: None)**								
Yes	0.319***	0.688***	0.425***	0.698***	0.320***	0.703***	0.304***	0.581***
	(0.0184)	(0.0460)	(0.0244)	(0.0478)	(0.0185)	(0.0463)	(0.0362)	(0.0987)
**Religion (Ref: No religion)**								
Christian	0.334***	0.438***	0.357***	0.463***	0.335***	0.446***	0.257***	0.312***
	(0.0432)	(0.0816)	(0.0491)	(0.0895)	(0.0437)	(0.0849)	(0.0563)	(0.0875)
Traditionalist/other	4.204***	1.165	4.183***	1.285	4.253***	1.189	3.024***	0.443*
	(0.942)	(0.357)	(0.980)	(0.408)	(0.965)	(0.370)	(0.906)	(0.203)
Islam	0.962	0.508***	0.971	0.542**	0.967	0.520***	0.760	0.276***
	(0.173)	(0.120)	(0.183)	(0.133)	(0.176)	(0.125)	(0.199)	(0.0886)
**Region (Ref: Western)**								
Central	1.505	1.551	1.451	1.551	1.566	1.618	0.935	1.025
	(0.539)	(0.593)	(0.525)	(0.598)	(0.565)	(0.619)	(0.398)	(0.486)
Greater Accra	0.365**	1.207	0.426*	1.264	0.366**	1.226	0.257***	0.952
	(0.160)	(0.602)	(0.189)	(0.639)	(0.160)	(0.606)	(0.133)	(0.580)
Volta	3.727***	2.521**	3.519***	2.523**	3.839***	2.562**	2.645**	2.269*
	(1.368)	(0.957)	(1.310)	(0.968)	(1.423)	(0.974)	(1.120)	(1.072)
Eastern	1.049	1.069	1.106	1.121	1.108	1.121	0.219***	0.342**
	(0.656)	(0.662)	(0.709)	(0.714)	(0.694)	(0.694)	(0.105)	(0.173)
Ashanti	0.593	0.756	0.574	0.704	0.612	0.778	0.590	1.021
	(0.239)	(0.328)	(0.234)	(0.307)	(0.247)	(0.337)	(0.280)	(0.519)
Brong-Ahafo	1.979*	1.815	2.033*	1.858	2.073**	1.880	1.052	1.172
	(0.721)	(0.695)	(0.753)	(0.720)	(0.760)	(0.721)	(0.433)	(0.529)
Northern	15.04***	10.85***	14.17***	10.45***	15.60***	11.13***	10.72***	14.60***
	(5.224)	(4.317)	(4.995)	(4.220)	(5.479)	(4.433)	(4.164)	(6.915)
Upper East	35.04***	24.95***	34.75***	25.16***	36.40***	25.63***	24.25***	20.44***
	(12.38)	(10.11)	(12.45)	(10.19)	(12.99)	(10.44)	(9.686)	(10.79)
Upper West	9.924***	5.629***	9.367***	5.565***	10.15***	5.724***	9.193***	6.131***
	(3.522)	(2.199)	(3.427)	(2.216)	(3.628)	(2.243)	(3.565)	(2.710)
**Residence (Ref: Urban)**								
Rural	6.647***	3.413***	5.898***	3.321***	6.636***	3.435***	7.658***	3.909***
	(1.035)	(0.789)	(0.934)	(0.801)	(1.036)	(0.791)	(1.477)	(1.002)
**Rent agreement (Ref: Owning)**								
Renting	0.189***	0.969	0.206***	0.998	0.188***	0.963	0.243***	0.824
	(0.0502)	(0.295)	(0.0609)	(0.331)	(0.0511)	(0.299)	(0.0446)	(0.166)
Rent free	0.377***	0.857	0.383***	0.855	0.380***	0.863	0.434***	0.764
	(0.0400)	(0.107)	(0.0415)	(0.109)	(0.0406)	(0.108)	(0.0643)	(0.157)
Perching/squatting	0.607	1.510	0.596	1.461	0.623	1.541	0.421	1.634
	(0.267)	(0.950)	(0.264)	(0.926)	(0.276)	(0.976)	(0.317)	(1.507)
**National welfare quintile (Ref: First)**								
Second	0.329***	0.799	0.344***	0.792	0.328***	0.794	0.244***	0.889
	(0.0441)	(0.146)	(0.0474)	(0.148)	(0.0445)	(0.146)	(0.0393)	(0.300)
Third	0.166***	0.661	0.176***	0.633*	0.164***	0.657	0.139***	1.155
	(0.0234)	(0.171)	(0.0256)	(0.167)	(0.0234)	(0.171)	(0.0246)	(0.503)
Fourth	0.0867***	0.470**	0.0995***	0.469**	0.0859***	0.468**	0.0599***	0.679
	(0.0132)	(0.162)	(0.0153)	(0.168)	(0.0131)	(0.163)	(0.0120)	(0.342)
Fifth	0.0391***	0.312**	0.0520***	0.313**	0.0379***	0.307**	0.0287***	0.693
	(0.00722)	(0.145)	(0.0106)	(0.149)	(0.00689)	(0.143)	(0.00628)	(0.471)
Total expenditure per day per adult^a^	0.242***	0.851	0.264***	0.875	0.240***	0.853	0.195***	0.492**
	(0.0235)	(0.194)	(0.0275)	(0.205)	(0.0236)	(0.197)	(0.0202)	(0.156)
Household size	1.154***	0.961**	1.134***	0.965**	1.154***	0.961**	1.169***	0.897***
	(0.0204)	(0.0165)	(0.0203)	(0.0166)	(0.0207)	(0.0166)	(0.0219)	(0.0300)
**FI2 (Ref: No financial inclusion)**								
Yes			0.960	1.019	0.215***	0.694***		
			(0.134)	(0.224)	(0.0181)	(0.0727)		
**FI3 (Ref: Informal financial inclusion)**								
Yes							0.207***	0.617*
							(0.0310)	(0.180)
Observations		36,433		30,737		35,737		6,460

Odds Ratios (ORs) are used; Standard errors in parentheses; FI1: Combined financial inclusion; FI2: Refers to informal financial inclusion in panel B but formal financial inclusion in panel C; FI3: Refers to formal financial inclusion; a: log of variable is used;

* *p* < 0.1,

** *p* < 0.05,

*** *p* < 0.01.

*Source*: Authors’ computation from GLSS7.

**Table 3:** Logistic regressions: Effect of financial inclusion on sharing of toilet facilities.

	Unadjusted ORs	(A) Adjusted ORs	Unadjusted ORs	(B) Adjusted ORs	Unadjusted ORs	(C) Adjusted ORs	Unadjusted ORs	(D) Adjusted ORs
**FI1 (Ref: No financial inclusion)**								
Yes	0.707***	0.798***						
	(0.0495)	(0.0498)						
**Sex (Ref: Female)**								
Male	1.061*	1.092**	1.045	1.072	1.058	1.093**	1.246***	1.257***
	(0.0379)	(0.0469)	(0.0449)	(0.0518)	(0.0387)	(0.0479)	(0.0771)	(0.0989)
**Educational qualification (Ref: None)**								
Yes	0.693***	0.803***	0.826***	0.852**	0.689***	0.807***	0.533***	0.574***
	(0.0380)	(0.0482)	(0.0480)	(0.0558)	(0.0374)	(0.0498)	(0.0624)	(0.0744)
**Religion (Ref: No religion)**								
Christian	0.503***	0.495***	0.489***	0.448**	0.491***	0.489***	0.698	0.717
	(0.112)	(0.130)	(0.123)	(0.144)	(0.110)	(0.129)	(0.190)	(0.226)
Traditionalist/other	0.436**	0.436*	0.403**	0.402*	0.419**	0.432*	0.696	0.577
	(0.153)	(0.195)	(0.158)	(0.208)	(0.147)	(0.194)	(0.305)	(0.292)
Islam	0.824	0.964	0.775	0.896	0.801	0.945	1.195	1.135
	(0.221)	(0.316)	(0.231)	(0.347)	(0.217)	(0.311)	(0.385)	(0.449)
**Region (Ref: Western)**								
Central	1.646***	1.617**	1.731***	1.703**	1.672***	1.631**	1.252	1.411
	(0.315)	(0.338)	(0.346)	(0.361)	(0.322)	(0.343)	(0.338)	(0.401)
Greater Accra	0.691*	0.723	0.793	0.739	0.685*	0.720	0.577*	0.703
	(0.151)	(0.185)	(0.181)	(0.202)	(0.149)	(0.186)	(0.167)	(0.209)
Volta	1.665**	1.522	1.656**	1.560	1.673**	1.539	1.630	1.288
	(0.351)	(0.412)	(0.359)	(0.425)	(0.352)	(0.419)	(0.517)	(0.412)
Eastern	1.581**	1.404*	1.663***	1.506**	1.593***	1.423*	1.283	1.137
	(0.284)	(0.279)	(0.313)	(0.305)	(0.287)	(0.285)	(0.350)	(0.311)
Ashanti	1.651***	1.334	1.936***	1.532*	1.686***	1.367	1.052	0.890
	(0.301)	(0.285)	(0.385)	(0.352)	(0.308)	(0.294)	(0.258)	(0.230)
Brong-Ahafo	1.770**	1.563*	1.734**	1.486	1.762**	1.556*	1.868**	1.867**
	(0.394)	(0.393)	(0.395)	(0.390)	(0.397)	(0.395)	(0.555)	(0.537)
Northern	1.066	1.079	1.028	1.084	1.058	1.097	1.415	1.272
	(0.350)	(0.399)	(0.355)	(0.396)	(0.356)	(0.409)	(0.523)	(0.913)
Upper East	0.726	0.543	0.759	0.597	0.697	0.531	0.720	0.474
	(0.256)	(0.214)	(0.261)	(0.227)	(0.251)	(0.210)	(0.333)	(0.264)
Upper West	0.572*	0.422**	0.533**	0.441**	0.554*	0.420**	1.051	0.585
	(0.177)	(0.165)	(0.170)	(0.170)	(0.172)	(0.165)	(0.434)	(0.310)
**Residence (Ref: Urban)**								
Rural	1.125	1.093	0.976	1.044	1.127	1.087	1.414**	1.278
	(0.130)	(0.149)	(0.117)	(0.149)	(0.130)	(0.149)	(0.221)	(0.207)
**Rent agreement (Ref: Owning)**								
Renting	3.184***	4.111***	3.632***	4.141***	3.159***	4.070***	3.671***	4.709***
	(0.387)	(0.521)	(0.507)	(0.609)	(0.385)	(0.514)	(0.444)	(0.653)
Rent free	2.486***	2.530***	2.417***	2.292***	2.461***	2.498***	3.269***	3.436***
	(0.298)	(0.332)	(0.301)	(0.315)	(0.296)	(0.329)	(0.493)	(0.568)
Perching/squatting	1.269	1.371	0.978	1.003	1.314	1.394	2.940	4.151
	(0.787)	(0.832)	(0.623)	(0.585)	(0.830)	(0.845)	(2.446)	(5.298)
**National welfare quintile (Ref: First)**								
Second	1.343*	1.580**	1.376**	1.399	1.333*	1.559**	1.259	1.755
	(0.222)	(0.341)	(0.224)	(0.308)	(0.224)	(0.340)	(0.342)	(0.632)
Third	1.295*	1.531*	1.325*	1.297	1.293	1.513*	1.244	1.667
	(0.202)	(0.350)	(0.211)	(0.342)	(0.204)	(0.346)	(0.284)	(0.583)
Fourth	1.066	1.514	1.143	1.195	1.060	1.484	0.973	1.987*
	(0.171)	(0.434)	(0.190)	(0.403)	(0.171)	(0.422)	(0.220)	(0.749)
Fifth	0.686**	1.310	0.786	0.951	0.684**	1.299	0.621**	2.025
	(0.106)	(0.490)	(0.127)	(0.428)	(0.106)	(0.478)	(0.134)	(0.955)
Total expenditure per day per adult^a^	0.783***	0.542***	0.899	0.646**	0.782***	0.547***	0.613***	0.368***
	(0.0606)	(0.0909)	(0.0768)	(0.132)	(0.0609)	(0.0905)	(0.0509)	(0.0711)
Household size	0.920***	0.888***	0.909***	0.902**	0.919***	0.887***	0.893***	0.830***
	(0.0239)	(0.0368)	(0.0267)	(0.0364)	(0.0243)	(0.0373)	(0.0238)	(0.0396)
**FI2 (Ref: No financial inclusion)**								
Yes			1.095	1.115	0.681***	0.772***		
			(0.171)	(0.209)	(0.0486)	(0.0496)		
**FI3 (Ref: Informal financial inclusion)**								
Yes							0.617***	0.678*
							(0.0989)	(0.142)
Observations		16,562		12,821		16,275		4,059

Odds Ratios (ORs) are used; Standard errors in parentheses; FI1: Combined financial inclusion; FI2: Refers to informal financial inclusion in panel B but formal financial inclusion in panel C; FI3: Refers to formal financial inclusion; a: log of variable is used;

* *p* < 0.1,

** *p* < 0.05,

*** *p* < 0.01.

*Source*: Authors’ computation from GLSS7.

Concerning the relationship between financial inclusion and open defecation (Table 2), we find that financial inclusion is associated with a decrease in the likelihood of open defecation. Specifically, individuals with financial inclusion are found to be associated with 0.75 times lesser odds of engaging in open defecation relative to those without financial inclusion (*p* < 0.01). Also, we find no statistically significant difference between those with informal financial inclusion and those without financial inclusion with regard to open defecation (*p*>0.1). However, having formal financial inclusion is found to be associated with 0.69 times (*p* < 0.01) and 0.62 times (*p* < 0.1) lower odds of engaging in open defecation relative to not having financial inclusion and having informal financial inclusion respectively.

As regards sex, in the formal financial inclusion vs informal financial inclusion model (Table 2, Panel D), males are found to be associated with 1.26 times higher odds of engaging in open defecation relative to females (*p* < 0.1). Also, individuals who have formal educational qualifications are less likely to engage in open defecation relative to those with no formal educational qualifications. Specifically, individuals with formal educational qualifications are associated with between 0.58 to 0.70 times (*p* < 0.01) lesser odds of engaging in open defecation relative to those without formal educational qualifications (Table 2).

As regards religion, Christians are found to be associated with 0.31 to 0.46 times (*p* < 0.01) lesser odds of engaging in open defecation as compared to those with no religion. Similarly, Muslims are associated with 0.28 to 0.54 times (*p* < 0.01 and *p* < 0.05) lesser odds of engaging in open defecation as compared to those with no religion (Table 2). Similar result is found between individuals who belong to Traditional or other religion and those with no religion (Table 2, Panel D).

Turning to region, our results show that, individuals in the Volta Region are associated with 2.27 to 2.56 times (*p* < 0.05 and *p* < 0.1) greater odds of engaging in open defecation relative to those in the Western Region. Similarly, individuals in the Northern, Upper East and Upper West Regions are associated with 10.45 to 14.60 times (*p* < 0.01), 20.44 to 25.63 times (*p* < 0.01) and 5.57 to 6.13 times (*p* < 0.01) higher odds of involving in open defecation respectively, relative to those in the Western Region (Table 2). Nonetheless, individuals in the Eastern Region are associated with 0.34 times (*p* < 0.05) lesser odds of engaging in open defecation as compared to those in the Western Region (Table 2, Panel D).

Concerning residence type, the results indicate that individuals in rural areas are associated with 3.32 to 3.91 times (*p* < 0.01) greater odds of engaging in open defecation relative to individuals in urban areas (Table 2).

Also, individuals in the 3^rd^, 4^th^ and 5^th^ national welfare quintiles are found to be associated with lesser odds of involving in open defecation relative to those in the least or 1^st^ national welfare quintile (Table 2, Panels A-C). Moreover, a unit increase in total adult expenditure per day is found to be associated with 0.49 times (*p* < 0.05) lesser odds of engaging in open defecation (Table 2, Panel D). Similarly, a unit increase in household size is found to be associated with 0.90 to 0.97 times (*p* < 0.01 and *p* < 0.05) lower odds of engaging in open defecation (Table 2).

In Table 3, with regard to the relationship between financial inclusion and sharing of toilet facilities among households, we find it to be negative. Specifically, we find that individuals with financial inclusion are associated with 0.80 times (*p* < 0.01) lesser odds of sharing a toilet facility with another household relative to those without financial inclusion. Also, we find no statistically significant difference between individuals with informal financial inclusion and those without financial inclusion concerning sharing of toilet facilities. However, individuals with formal financial inclusion are found to be associated with 0.77 times (*p* < 0.01) and 0.68 times (*p* < 0.1) lesser odds of sharing toilet facilities with other households relative to those without financial inclusion and those with informal financial inclusion respectively.

Also, males are found to be associated with 1.09 to 1.26 times (*p* < 0.01 and *p* < 0.05) greater odds of sharing a toilet facility with other households relative to females. Nonetheless, having formal educational qualification is found to be associated with 0.57 to 0.85 times (*p* < 0.01 and *p* < 0.05) lower odds of sharing a toilet facility with other households (Table 3).

With regard to religion, Christians and individuals who belong to Traditional/other religions are found to be associated with 0.45 to 0.50 times (*p* < 0.01 and *p* < 0.05) and 0.40 to 0.44 times (*p* < 0.1) lesser odds of sharing toilet facilities with other households respectively, relative to those without any religion (Table 3, Panels A-C).

On regional basis, relative to individuals in the Western Region, we find that those in the Central Region are associated with 1.62 to 1.70 times (*p* < 0.05) higher odds of sharing toilet facilities with other households. Similarly, individuals in the Eastern and Brong Ahafo Regions are associated with 1.40 to 1.51 times (*p* < 0.05 and *p* < 0.1) and 1.56 to 1.87 times (*p* < 0.05 and *p* < 0.1) higher odds of sharing toilet facilities with other households respectively, relative to those in the Western Region. However, individuals in the Upper West Region are associated with 0.42 to 0.44 times (*p* < 0.05) lesser odds of sharing toilet facilities with other households relative to those in the Western Region (Table 3).

Concerning rent agreement or ownership status of dwelling, we find that individuals who are in rented dwellings and those in rent free houses are associated with 4.07 to 4.71 times (*p* < 0.01) and 2.29 to 3.44 times (*p* < 0.01) higher odds of sharing toilet facilities with other households respectively, relative to those who own their dwellings. Nonetheless, we find that individuals in the least (1^st^) national welfare quintile (who have toilets) are associated with a lesser likelihood to share toilet facilities with other households relative to those in the 2^nd^, 3^rd^ and 4^th^ national welfare quintile. Further, total expenditure in a day per adult and household size are found to be associated with 0.37 to 0.65 times (*p* < 0.01 and *p* < 0.05) and 0.83 to 0.90 times (*p* < 0.01 and *p* < 0.05) lesser odds of sharing toilet facilities with other households respectively (Table 3).

### Robustness checks

To check the robustness of our estimates, we use the binary probit regression as an alternative estimation technique. We find that the results are qualitatively similar. Specifically concerning the main variable of interest; financial inclusion, we find that it is negatively associated with open defecation and sharing of toilet facilities among households. Moreover, while there is no statistically significant difference between those with informal financial inclusion and those without financial inclusion, having formal financial inclusion is found to be associated with lesser likelihood of open defecation and sharing of toilet facilities relative to not having financial inclusion and having informal financial inclusion (see Appendix).

## Discussion

This study investigates how financial inclusion is associated with open defecation and sharing of toilet facilities among households in Ghana whiles controlling for factors such as education, sex, region, religion, residence type, rent agreement, national welfare quintile, total expenditure in a day per adult and household size.

The findings point to the fact that financial inclusion in general, can play a very important role in improving open defecation as well as sharing of toilet facilities among households. This outcome is not surprising since financial inclusion can make financial resources easily accessible to individuals/households and hence bolster their ability to afford toilet facilities. Moreover, in Ghana, since a lot of people use public toilets at a fee, financial inclusion can enhance people’s ability to afford such fees than to opt for open defecation. Our results on the statistically insignificant difference between those without financial inclusion and those with informal financial inclusion can be attributed to the fact that, most informal financial inclusion schemes are unable to provide higher financial resources relative to formal ones. Moreover, informal financial inclusion schemes such as susu in Ghana, normally provide financial resources to individuals in turns, and the likelihood of receiving is highly dependent on other members of the scheme paying their contributions. Our finding conflicts with those of Ezenwaji and Otti [23] and Barenberg et al. [25] who found informal financial inclusion to enhance access to sanitation facilities in Nigeria and India, respectively. The difference with our findings could be that, unlike our informal financial inclusion indicator, the informal financial inclusion indicator used by these studies were loans given specifically to acquire sanitation facilities.

However, comparing formal and informal financial inclusion, our findings show that, having formal financial inclusion, is associated with lesser likelihood of open defecation and sharing of toilet facilities among households relative to not having financial inclusion and having informal financial inclusion. These outcomes are not surprising since individuals with formal financial inclusion are more likely to have access to greater financial resources relative to those with informal financial inclusion or without financial inclusion. Thus, to enhance open defecation and sharing of toilet facilities among households, attention should be paid towards enhancing formal financial inclusion. Similar outcomes have been found in India [22] and Kenya [24] with regard to the role of formal financial inclusion in enhancing access to sanitation facilities.

The finding on males being more likely to be associated with open defecation could be linked to the traditional/cultural thinking that men can urinate or defecate anywhere relative to women. Indeed, previous studies by Osumanu et al. [18] and Adjibolosoo et al. [19] found traditional norms to influence open defecation in Ghana. The finding on males is similar to that of Adams et al. [14] but conflicts with the findings of Mulenga et al. [8] and Akpakli et al. [15] with regard to access to improved sanitation facilities or services.

The negative association between education on hand, and open defecation and sharing of toilet facilities among households on another hand is not farfetched because, education can make individuals better understand the health benefits of not defecating in open places as well as sharing toilet facilities. In fact, education has been revealed to be associated with the utilisation of health enhancing inputs [31–35]. Moreover, individuals with educational qualifications are more likely to earn higher [36], hence making them more capable of affording toilet facilities for their households. This outcome is similar to the findings of Mosimane and Kamwi [13], Adams et al. [14], Akpakli et al. [15] as well as Adzawla et al. [20].

As regards religion, with Christians and Muslims both having the doctrine of ‘cleanliness is next to Godliness’ it is not surprising that they are less probable to practice open defecation relative to those without any religion. Moreover, Christians and Muslims may consider open defecation as a sin. In fact, Bhatt et al. [10] also found in Nepal that religious beliefs affect open defecation.

There are regional differences with regard to open defecation and sharing of toilet facilities among households in Ghana. For instance, the finding on individuals in the Volta, Northern, Upper East and Upper West Regions being more likely to engage in open defecation is not surprising since according to the Ghana Statistical Service [37], these regions are some of the poorest areas in Ghana. Hence, individuals in these regions may be less capable of affording toilet facilities. Our finding concurs with Adams et al. [14] who found households in the Northern, Upper East and Upper West Regions in Ghana to be less likely to have access to improved sanitation facilities or services.

As regards residence type, since residents of urban areas are more likely to be richer and hence more capable of affording toilet facilities relative to rural dwellers, it is not farfetched that rural residents are found to be more likely to engage in open defecation. In fact, statistics show that a significant number of people engaged in open defecation are in rural areas [2,38]. Similar finding was reported by Adzawla et al. [20].

The finding on individuals in rented and rent-free dwellings being associated with sharing toilet facilities relative to those who own their dwellings is not surprising. This is because those who own their dwellings are more capable of affording toilet facilities and also would not need the permission of any landlord or landlady to install such facilities relative to those who have rented or are in rent free dwellings.

Also, as expected, rising adult expenditure is found to be associated with lesser likelihood of open defecation and sharing of toilet facilities. Thus, people who spend more are likely to have the financial resources to acquire toilet facilities. Also, the outcome with regard to individuals in the least national welfare quintile being more likely to practice open defecation is not farfetched because, such individuals are most likely to be poor, hence may not be able to afford toilet facilities.

Last but not the least, the implication of the finding on rising household size being associated with lesser likelihood of open defecation and sharing a toilet facility could be that, as the number of household members increases, households may be more prepared to get their own toilet facilities because sharing with another household may be highly inconvenient and inadequate. Higher household size could also mean more pooled financial resources from individual members, which can increase the ability to afford toilet facilities. Similar outcome was reported by Adzawla et al. [20] with regard to household size increasing the preference for public toilets over open defecation in Ghana. Nonetheless, our result conflicts with Osumanu et al. [18] who found household size to increase open defecation in the Wa Municipality of Ghana.

## Conclusion

Open defecation and sharing of toilet facilities among households are known to be associated with numerous negative health effects. Meanwhile, globally and in Ghana, many people do practice open defecation as well as share toilet facilities with other households. This has resulted in a number of studies devoted to the determinants of access to sanitation facilities among households in Ghana. Nonetheless, while financial inclusion is deemed to be a major enabler of social welfare, to the best of our knowledge, no empirical study has been devoted to the relationships between financial inclusion, and open defecation as well as sharing of toilet facilities among households in Ghana. This study therefore uses datasets from the GLSS7 to examine how financial inclusion is associated with open defecation and sharing of toilet facilities among households in Ghana. The binary logit regression is used as the empirical estimation technique. We find that financial inclusion in general, is negatively associated with open defecation and sharing of toilet facilities among households in Ghana. Looking at formal and informal financial inclusion, our results show that, while informal financial inclusion is statistically insignificant, having formal financial inclusion is associated with reduced open defecation and sharing of toilet facilities among households.

The implication is that, as government of Ghana strives to eliminate open defecation as well as reduce the sharing of toilet facilities among households, conscious efforts should be devoted towards enhancing formal financial inclusion.

## Appendices

**Table A1:** Probit regressions: Effect of financial inclusion on open defecation (robustness checks).

	Open defecation (A)	Open defecation (B)	Open defecation (C)	Open defecation (D)
**FI1 (Ref: No financial inclusion)**				
Yes	-0.15***			
	(0.05)			
**Sex (Ref: Female)**				
Male	0.01	0.01	0.02	0.12*
	(0.02)	(0.02)	(0.02)	(0.06)
**Educational qualification (Ref: None)**				
Yes	-0.21***	-0.20***	-0.19***	-0.29***
	(0.04)	(0.04)	(0.03)	(0.09)
**Religion (Ref: No religion)**				
Christian	-0.46***	-0.43***	-0.45***	-0.60***
	(0.10)	(0.11)	(0.10)	(0.15)
Traditionalist/other	0.11	0.17	0.12	-0.41*
	(0.17)	(0.18)	(0.17)	(0.23)
Islam	-0.41***	-0.38***	-0.40***	-0.71***
	(0.13)	(0.13)	(0.13)	(0.17)
**Region (Ref: Western)**				
Central	0.26	0.26	0.29	0.06
	(0.20)	(0.21)	(0.20)	(0.23)
Greater Accra	0.12	0.14	0.14	0.03
	(0.24)	(0.25)	(0.24)	(0.27)
Volta	0.50**	0.50**	0.51**	0.43*
	(0.20)	(0.21)	(0.20)	(0.24)
Eastern	0.02	0.04	0.04	-0.43*
	(0.32)	(0.33)	(0.32)	(0.23)
Ashanti	-0.15	-0.19	-0.13	0.01
	(0.22)	(0.22)	(0.22)	(0.24)
Brong-Ahafo	0.31	0.32	0.33	0.10
	(0.21)	(0.21)	(0.21)	(0.22)
Northern	1.38***	1.37***	1.40***	1.47***
	(0.22)	(0.22)	(0.22)	(0.24)
Upper East	1.86***	1.87***	1.87***	1.69***
	(0.22)	(0.22)	(0.22)	(0.26)
Upper West	1.00***	1.00***	1.01***	1.01***
	(0.21)	(0.22)	(0.21)	(0.22)
**Residence (Ref: Urban)**				
Rural	0.65***	0.64***	0.65***	0.66***
	(0.12)	(0.12)	(0.12)	(0.12)
**Rent agreement (Ref: Owning)**				
Renting	-0.02	-0.01	-0.03	-0.08
	(0.14)	(0.16)	(0.14)	(0.10)
Rent free	-0.07	-0.08	-0.07	-0.12
	(0.07)	(0.07)	(0.07)	(0.10)
Perching/squatting	0.30	0.28	0.31	0.40
	(0.36)	(0.36)	(0.36)	(0.49)
**National welfare quintile (Ref: First)**				
Second	-0.14	-0.14	-0.14	-0.16
	(0.10)	(0.10)	(0.10)	(0.17)
Third	-0.23	-0.25*	-0.23	-0.04
	(0.14)	(0.15)	(0.14)	(0.21)
Fourth	-0.40**	-0.40**	-0.40**	-0.33
	(0.18)	(0.20)	(0.19)	(0.24)
Fifth	-0.57**	-0.58**	-0.58**	-0.33
	(0.24)	(0.26)	(0.25)	(0.32)
Total expenditure per day per adult^a^	-0.12	-0.10	-0.12	-0.33**
	(0.12)	(0.13)	(0.12)	(0.14)
Household size	-0.02**	-0.02**	-0.02**	-0.06***
	(0.01)	(0.01)	(0.01)	(0.02)
**FI2 (Ref: No financial inclusion)**				
Yes		0.02	-0.19***	
		(0.12)	(0.05)	
**FI3 (Ref: Informal financial inclusion)**				
Yes				-0.28*
				(0.15)
Constant	-0.63**	-0.70**	-0.67**	0.25
	(0.32)	(0.34)	(0.32)	(0.36)
Observations	36433	30737	35737	6460

Coefficients are used; Standard errors in parentheses; FI1: Combined financial inclusion; FI2: Refers to informal financial inclusion in panel B but formal financial inclusion in panel C; FI3: Refers to formal financial inclusion; a: Log of variable is used;

* *p* < 0.1,

** *p* < 0.05,

*** *p* < 0.01.

*Source*: Authors’ computation from GLSS7.

**Table A2:** Probit regressions: Effect of financial inclusion on sharing of toilet facilities (robustness checks).

	Sharing (A)	Sharing (B)	Sharing (C)	Sharing (D)
**FI1 (Ref: No financial inclusion)**				
Yes	-0.14***			
	(0.04)			
**Sex (Ref: Female)**				
Male	0.05**	0.04	0.05**	0.14***
	(0.03)	(0.03)	(0.03)	(0.05)
**Educational qualification (Ref: None)**				
Yes	-0.13***	-0.09**	-0.13***	-0.32***
	(0.04)	(0.04)	(0.04)	(0.08)
**Religion (Ref: No religion)**				
Christian	-0.41***	-0.46**	-0.41***	-0.19
	(0.15)	(0.19)	(0.15)	(0.19)
Traditionalist/other	-0.48*	-0.52*	-0.49*	-0.34
	(0.27)	(0.31)	(0.27)	(0.31)
Islam	-0.01	-0.04	-0.02	0.08
	(0.19)	(0.22)	(0.19)	(0.24)
**Region (Ref: Western)**				
Central	0.29**	0.32**	0.29**	0.20
	(0.13)	(0.13)	(0.13)	(0.17)
Greater Accra	-0.20	-0.18	-0.20	-0.22
	(0.16)	(0.17)	(0.16)	(0.18)
Volta	0.25	0.27	0.26	0.15
	(0.16)	(0.16)	(0.16)	(0.19)
Eastern	0.21*	0.25**	0.22*	0.07
	(0.12)	(0.12)	(0.12)	(0.17)
Ashanti	0.17	0.26*	0.19	-0.08
	(0.13)	(0.14)	(0.13)	(0.16)
Brong-Ahafo	0.26*	0.23	0.26*	0.37**
	(0.15)	(0.16)	(0.15)	(0.17)
Northern	0.05	0.05	0.06	0.15
	(0.22)	(0.22)	(0.22)	(0.40)
Upper East	-0.38	-0.32	-0.39	-0.46
	(0.24)	(0.23)	(0.24)	(0.34)
Upper West	-0.52**	-0.50**	-0.52**	-0.30
	(0.24)	(0.23)	(0.24)	(0.31)
**Residence (Ref: Urban)**				
Rural	0.06	0.03	0.05	0.15
	(0.08)	(0.09)	(0.08)	(0.10)
**Rent agreement (Ref: Owning)**				
Renting	0.87***	0.87***	0.86***	0.95***
	(0.08)	(0.09)	(0.08)	(0.08)
Rent free	0.56***	0.50***	0.56***	0.75***
	(0.08)	(0.08)	(0.08)	(0.10)
Perching/squatting	0.21	-0.00	0.22	0.86
	(0.38)	(0.38)	(0.38)	(0.66)
**National welfare quintile (Ref: First)**				
Second	0.29**	0.21	0.28**	0.36*
	(0.13)	(0.13)	(0.13)	(0.21)
Third	0.26*	0.16	0.26*	0.31
	(0.14)	(0.16)	(0.14)	(0.20)
Fourth	0.25	0.11	0.24	0.41*
	(0.17)	(0.21)	(0.17)	(0.22)
Fifth	0.16	-0.03	0.16	0.42
	(0.23)	(0.27)	(0.22)	(0.28)
Total expenditure per day per adult^a^	-0.37***	-0.27**	-0.36***	-0.58***
	(0.10)	(0.12)	(0.10)	(0.11)
Household size	-0.07***	-0.06***	-0.07***	-0.10***
	(0.02)	(0.02)	(0.02)	(0.03)
**FI2 (Ref: No financial inclusion)**				
Yes		0.07	-0.16***	
		(0.11)	(0.04)	
**FI3 (Ref: Informal financial inclusion)**				
Yes				-0.23*
				(0.13)
Constant	1.26***	1.14***	1.26***	1.79***
	(0.26)	(0.27)	(0.26)	(0.39)
Observations	16562	12821	16275	4059

Coefficients are used; Standard errors in parentheses; FI1: combined financial inclusion; FI2: Refers to informal financial inclusion in panel B but formal financial inclusion in panel C; FI3: Refers to formal financial inclusion; a: Log of variable is used;

* *p* < 0.1,

** *p* < 0.05,

*** *p* < 0.01.

*Source*: Authors’ computation from GLSS7.

## Supporting information

S1 File (ZIP)
